# Nitrous oxide as an effective AFM tip functionalization: a comparative study

**DOI:** 10.3762/bjnano.10.30

**Published:** 2019-01-30

**Authors:** Taras Chutora, Bruno de la Torre, Pingo Mutombo, Jack Hellerstedt, Jaromír Kopeček, Pavel Jelínek, Martin Švec

**Affiliations:** 1 Regional Centre of Advanced Technologies and Materials, Department of Physical Chemistry, Faculty of Science, Palacký University, Šlechtitelů 27, 78371 Olomouc, Czech Republic; 2 Institute of Physics of the Czech Academy of Sciences, Cukrovarnická 10, 162 00 Prague, Czech Republic

**Keywords:** atomic force microscopy, Au(111), carbon monoxide, functionalization, high resolution, nitrous oxide, submolecular resolution

## Abstract

We investigate the possibility of functionalizing Au tips by N_2_O molecules deposited on a Au(111) surface and their further use for imaging with submolecular resolution. First, we characterize the adsorption of the N_2_O species on Au(111) by means of atomic force microscopy with CO-functionalized tips and density functional theory (DFT) simulations. Subsequently we devise a method of attaching a single N_2_O to a metal tip apex and benchmark its high-resolution imaging and spectroscopic capabilities using FePc molecules. Our results demonstrate the feasibility of high-resolution imaging. However, we find an inherent asymmetry of the N_2_O probe-particle adsorption on the tip apex, in contrast to a CO tip reference. These findings are consistent with DFT calculations of the N_2_O- and CO tip apexes.

## Introduction

Frequency-modulated atomic force microscopy (AFM) has become the tool of choice for the characterization of molecules on the atomic scale. Functionalization of a metallic tip apex with a single carbon monoxide molecule (CO) was the key to achieve submolecular resolution for the first time, on a pentacene molecule [1]. This milestone initiated a vigorous development of the technique that now serves a variety of purposes. For example, it can identify molecular structures of natural and pure compounds [2–5], determine the bond order in conjugated systems [6], visualize intramolecular charge distributions [7–9], image three-dimensional molecular structures [10–12], discern complex molecular mixtures [13–14], resolve the intermediate states of chemical reactions [15–19] or discriminate the spin state of single molecules [20].

In most of these cases, the functionalized tip is routinely obtained by picking up a single CO molecule from the substrate. Applying an analogous approach, atomically sharp metal apexes can be also decorated either by different molecular species such as C_60_ [21], naphthalenetetracarboxylic diimide (NTCDI) [22], NO [23] or single atoms such as Xe [24–25], Br [24], Kr [24], O [26], and Cl [1,27]. Such tip terminations have proved to be fairly stable and therefore capable of achieving submolecular resolution. The characteristics of each type of tip termination, such as chemical structure or internal charge distribution, are extremely important for the AFM contrast, distortions in the molecule images, and spatial resolution [8,27–28]. The tip-terminating particle also significantly affects the spectroscopy measurements, i.e., the interaction energy toward different atomic species in force spectroscopy, the contact potential difference in Kelvin probe force microscopy (KPFM) [9,29] and vibrational levels of inelastic tunneling spectroscopy (IETS) [30–31]. A particular termination of the tip may be bound to certain types of substrates, and better suited for a limited range of investigated objects, such as molecules with specific functional groups or atomic impurities with characteristic charge distribution. Therefore it is of utmost importance to search for new potentially practical molecules for tip functionalization and describe their unique properties.

Here we present a process in which N_2_O was deposited on a Au(111) substrate and characterized. Subsequently we functionalized the Au tip with N_2_O and benchmarked its capabilities by imaging a FePc molecule and performing force–distance spectroscopy. The data is compared to equivalent measurements done with a Au tip functionalized with CO.

## Results and Discussion

**Figure 1:**
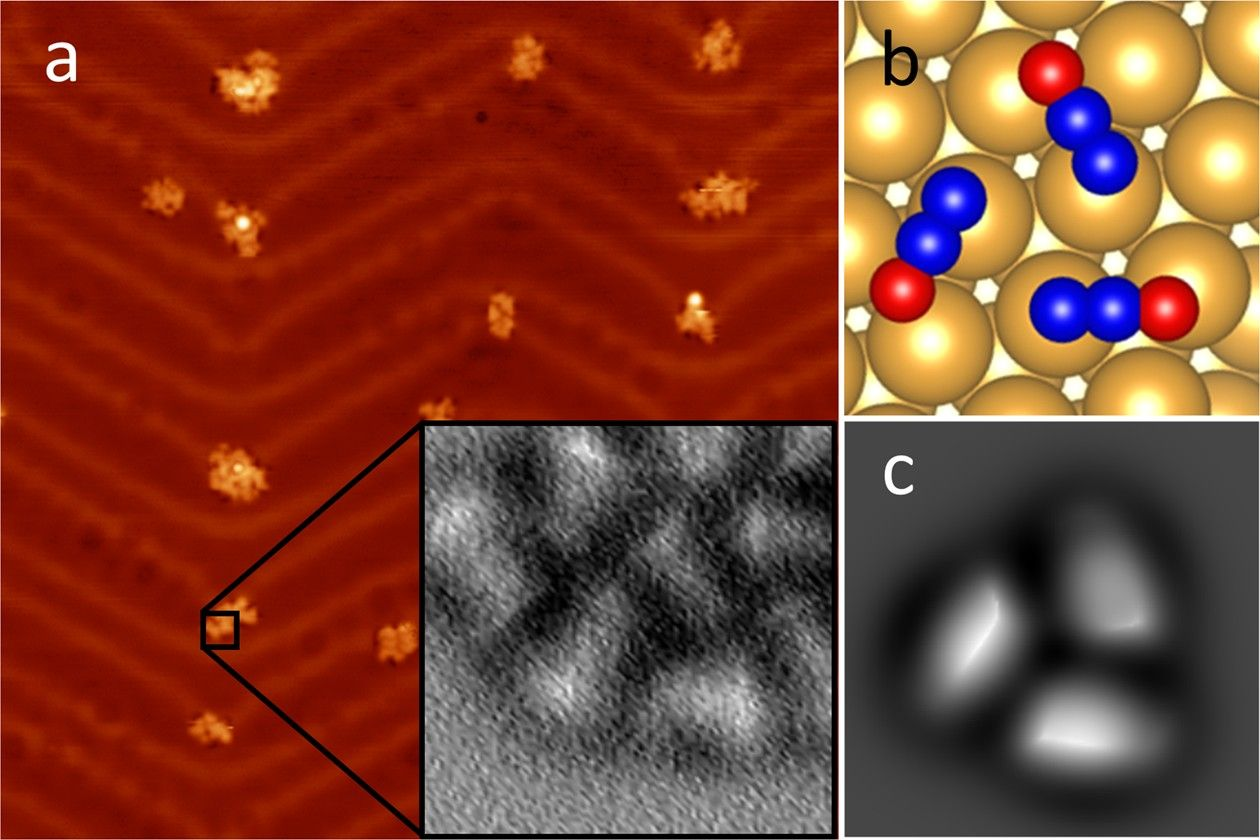
Adsorption of N_2_O molecules on the Au(111) substrate. (a) Overview STM image (100 mV, 10 pA, 50 × 50 nm^2^) of a sample after N_2_O deposition. Inset: a close-up AFM image (1.5 × 1.5 nm^2^) of the N_2_O cluster adsorbed on the herringbone elbow, scanned with a CO-functionalized tip. (b) Top view of the calculated adsorption geometry of a N_2_O trimer. (c) Simulated AFM image (1.5 × 1.5 nm^2^) of a N_2_O trimer on Au (111) using the probe-particle model [32].

A clean Au(111) surface was inserted into the microscope head and cooled to 5 K before exposing it to N_2_O gas. Figure 1a shows a characteristic constant-current image of the N_2_O/Au(111) system, revealing the formation of small 2D clusters, preferentially located at the kinks of the characteristic herringbone structure. Their variable size is typically a few nanometers in diameter. The estimated average apparent height of the cluster formations was 70 pm.

After the N_2_O cluster formation, the metallic tip (pre-treated by a gentle indentation into the substrate) was functionalized by an impurity CO molecule, which significantly improved the resolution in both STM and AFM. We performed high-resolution AFM/STM measurements on various clusters (comparable to the inset of Figure 1a), which revealed elongated structures; we attribute these to individual flat-lying N_2_O molecules. In a cluster, typically composed of 5–25 molecules, the N_2_O molecules have a preferential short-range arrangement of rotationally symmetrical trimers, with intermolecular distances of about 4.3 Å. A DFT calculation of a single N_2_O molecule on the surface confirms that its adsorption configuration on Au(111) is primarily driven by a non-covalent dispersion interaction and prefers to orient its longer axis parallel to the 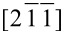 axis of the surface. The vertical distance between the single molecule and the surface was estimated to be 3.5 Å. Based on this finding, we construct an atomic model of the three flat-lying N_2_O molecules on Au(111) and optimize it with total-energy DFT calculations. We find that the trimer is stabilized by electrostatic interactions between the N and O atoms of adjacent N_2_O molecules, due to their slightly different polarization. The calculations reveal that the preferred orientation of the N_2_O molecules in the clusters is with the O atoms outward (Figure 1b), being 17 meV more stable than the opposite arrangement.

Using the optimized geometry of the cluster obtained from DFT calculations, as an input for the probe-particle model [32], we simulated the AFM images to determine the atomic contrast of the N_2_O trimer (Figure 1c). Note that the probe–particle was mimicking a CO molecule. We found good agreement between theory and experiment.

**Figure 2:**
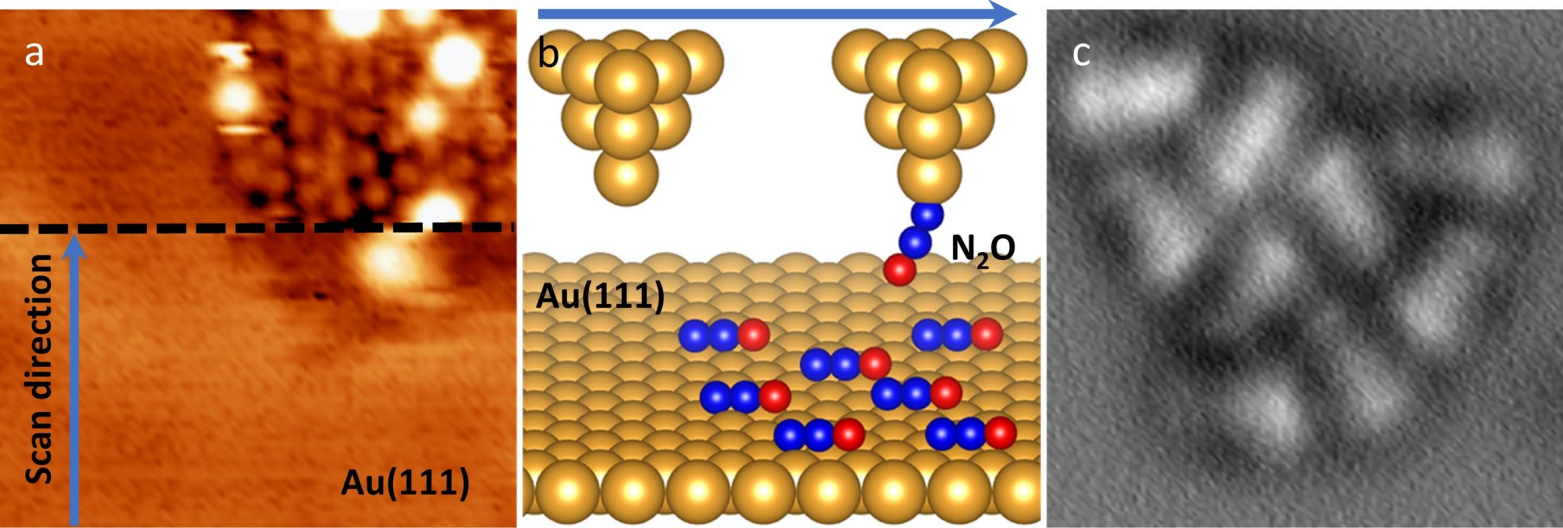
Tip functionalization with a N_2_O molecule. (a) STM image (100 mV, 10 pA, 6 × 6 nm^2^) demonstrating a spontaneous enhancement of the resolution while scanning over the N_2_O-covered surface. (b) Schematic representation of the functionalization process (blue arrow indicates the scan direction). (c) Constant-height AFM image (2 × 2 nm^2^) of a single N_2_O cluster obtained with a N_2_O-functionalized tip.

We were able to functionalize the tip with a N_2_O molecule. In various attempts to adsorb N_2_O onto the tip, we discovered that by intentionally reducing the bias to 50–100 mV for several seconds in constant-current mode while scanning an area containing a cluster of N_2_O molecules, a sudden improvement of the resolution occurred (as shown in Figure 2a). This event is characteristic for the tip picking up a molecule from the surface [33–34] and therefore can be attributed to a transfer of a N_2_O molecule from the surface to the tip apex, as schematically shown in Figure 2b. We propose that the N_2_O molecule is attached to the tip apex through the terminal N (Figure 2b), which has a more reactive character compared to the O atom [35]. In this manner, the O atom would be responsible for the majority of interaction with the substrate.

After functionalization of the tip apex with a single N_2_O molecule, we obtained a high-resolution AFM image of the N_2_O cluster (Figure 2c). The N_2_O tip exhibits good stability during the measurement, allowing us to scan at smaller tip–sample separations and to enter the Pauli repulsion regime. The AFM image of the N_2_O cluster Figure 2c shows a remarkably similar resolution to the images acquired with a CO-decorated tip.

**Figure 3:**
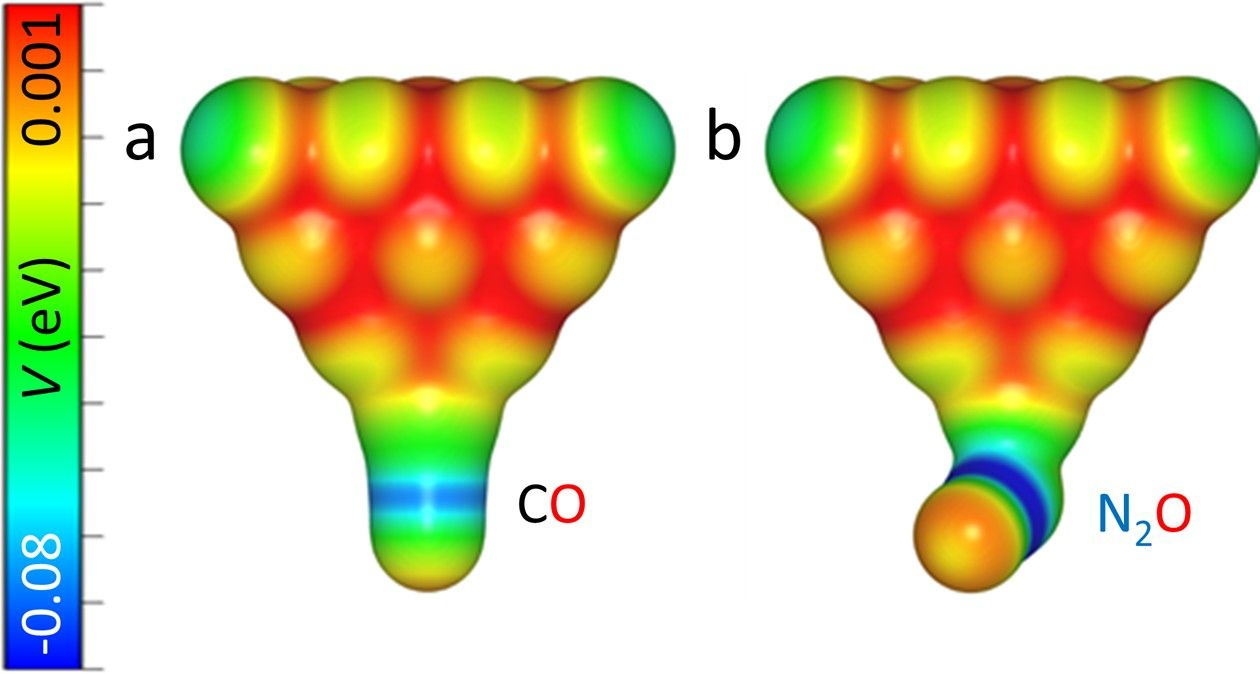
Comparison of the calculated electrostatic potential projections of the CO (a) and N_2_O (b) tips obtained through DFT calculations.

In order to understand the chemical behavior of the N_2_O tips and compare them to the CO tips, we carried out DFT calculations of their electrostatic potential and total densities (see Methods for more detail). Figure 3 shows the calculated electrostatic potential (ESP) map for CO and N_2_O attached to a Au pyramid, projected onto isosurfaces of their respective total electron densities (cut at 0.03 e/A^3^). The spatial ESP variation is an important factor for the determination of the molecular reactivity and can be interpreted as the static distribution of the charge around the molecule [36].

The ESP maps of the CO and the N_2_O molecule attached to the gold tip (Figure 3) possess some similar characteristics. Both molecules have similar variation of the potential along the probe molecule, i.e., the regions with negative values around the C–O and N–O bonds (electron-rich area, colored in blue) and regions with positive values at the terminal O atoms (electron-poor area, colored in red). This indicates that the N_2_O tip is very similar to the CO tip in terms of spatial charge distribution. However, the Hirschfeld analysis [37] of atomic charge at the O apex atom gives −0.077*e* for the N_2_O tip, compared to −0.055*e* for the CO tip. This can result in a larger electrostatic interaction of the N_2_O probe with a charged atom or molecule. Also, the geometry of the probe particles on the tip is remarkably different. The CO molecule is attached to the Au pyramid almost perfectly on its axis, whereas N_2_O is bent strongly. The bent adsorption configuration of the N_2_O molecule is caused by electrostatic interactions between the molecule and the Au tip, which arise from the mutual dipole–dipole interaction. Furthermore, the calculated adsorption energies of the two molecules on the tip differ as well. We have found a value of −0.840 eV for CO, compared to −0.156 eV for N_2_O. So while a N_2_O tip might still provide the resolution and sensitivity needed for submolecular imaging, an asymmetry is expected in the images made by the N_2_O tips and interaction forces may have a larger electrostatic contribution.

**Figure 4:**
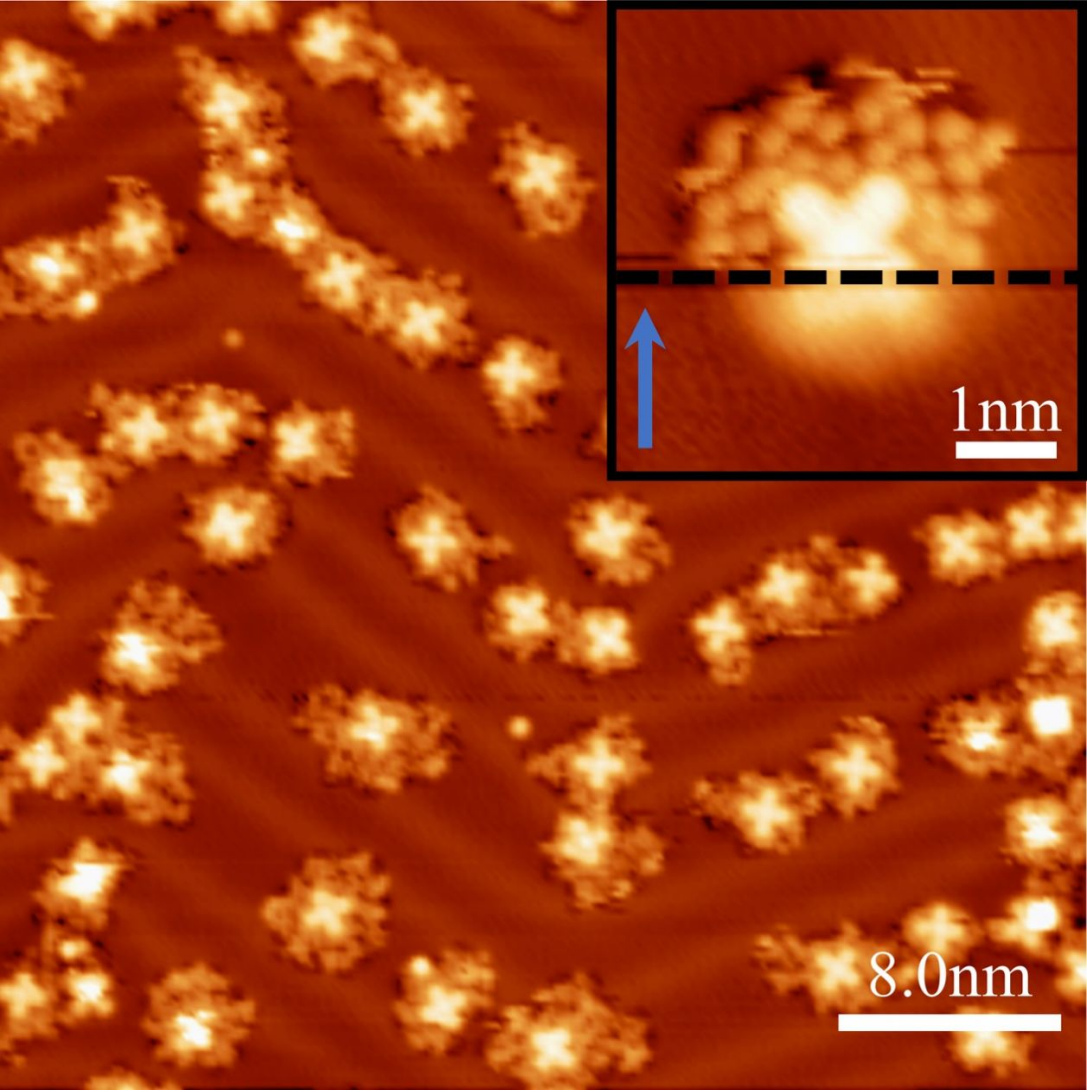
Constant-current STM images of the co-adsorption of FePc and N_2_O molecules on a Au(111) surface (200 mV, 20 pA, 40 × 40 nm^2^), imaged with a N_2_O-functionalized tip. Inset: STM image (50 mV, 20 pA, 5 × 5 nm^2^) of a FePc molecule surrounded by N_2_O species, demonstrating a tip-functionalization event (on the scan line marked by the dashed line). The scan direction is indicated by a blue arrow.

To benchmark the performance of the N_2_O-decorated tip experimentally, we used it to obtain high-resolution STM/AFM images of a single FePc molecule, which is suitable as a standard due to its planar shape and the flat adsorption geometry on Au(111) [31]. A submonolayer coverage of FePc molecules was deposited on Au(111) at room temperature, and the FePc/Au(111) surface was subsequently cooled down in the microscope and exposed to N_2_O. Figure 4 shows an overview STM image of the obtained sample, where the FePc molecules predominantly occupy the fcc-stacked Au regions and the kinks of the Au(111) herringbone reconstruction. The N_2_O species adsorbs planarly as in the previous experiment, clustering in the vicinity of single FePc molecules. To functionalize the tip with a single N_2_O molecule on such a sample we used the procedure described above. We scan a small region around a single FePc molecule that is surrounded by N_2_O molecules, at a setpoint of 50 mV and 20 pA until the characteristic change in the contrast, which is associated with the functionalization, occurs (inset of Figure 4).

**Figure 5:**
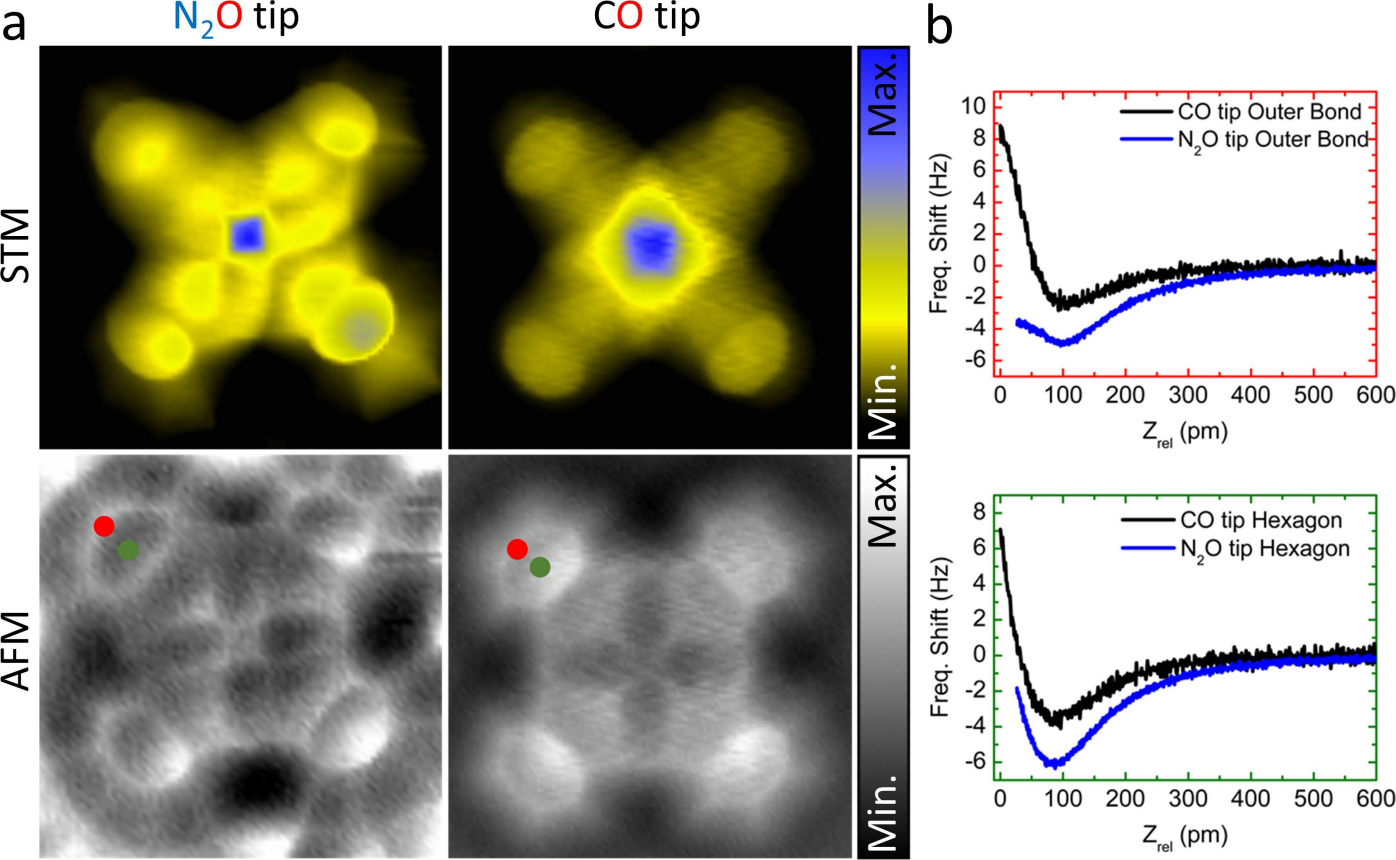
(a) STM and AFM constant-height images of the FePc on Au(111) (1.7 × 1.7 nm^2^, *V*_b_ = 3 mV) obtained with two different tip terminations, N_2_O and CO. The STM scale for N_2_O ranges from 0.6 to 43 pA and for CO from 3.8 to 410 pA. The AFM gray scale for N_2_O ranges from −23 to −12 Hz and for CO from −12 to 10 Hz. (b) Site-specific Δ*f* spectroscopy obtained with N_2_O and CO tip terminations above the outer C–C bonds (red dot) and the centers (green dot) of the peripheral benzene rings of the molecule.

With this functionalized tip, we performed imaging with submolecular resolution on one of the FePc molecules, surrounded by the N_2_O species. Figure 5a shows the corresponding set of constant-height STM/AFM maps, along with the reference data acquired with a CO tip on a single FePc molecule on Au(111). The observed AFM contrast for both the tips generally corresponds to the FePc backbone structure; it shows the four peripheral benzene rings, the inner pyrrole groups and a signature of the metal atom at the center. In the STM images both tips detect a dominating electron tunneling contribution of the central Fe molecular orbital at the Fermi level [31] and also the overall shape of the molecule.

The AFM image taken with the N_2_O tip exhibits slightly lower resolution, in comparison to the CO tip termination, with a strong directionality of the submolecular features within the peripheral benzene rings. The tunneling current image also reveals a significant shadow cast in the same direction as the asymmetric features in AFM. These features are indicative of a general probe asymmetry, consistent with the theoretical calculations, which shows a strongly bent adsorption configuration of the N_2_O molecule on the tip apex.

For a quantitative comparison of the interaction energy of the two tip terminations with FePc, we performed site-specific frequency-shift spectroscopy Δ*f*(*z*) measurements on the outer C–C bonds and centers of the peripheral benzene molecules indicated by the red and green dots in Figure 5a. In Figure 5b, the short-range Δ*f* curves recorded with N_2_O and CO tips are shown (after subtracting the background measured on clean Au [38]). The Δ*f*(*z*) dependence recorded for the N_2_O tips is considerably different from the one obtained with a CO tip, both qualitatively and quantitatively. The value of the maximum attractive force [39] for the N_2_O tip (Figure S1, Supporting Information File 1) on both spectroscopy sites (outer C–C bond, 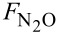 ≈ −125 pN, and hollow site, 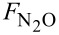 ≈ −132 pN) are significantly higher in comparison to the CO tip (outer C–C bond, *F*_CO_ ≈ −30 pN, and hollow site, *F*_CO_ ≈ −56 pN). Consequently, the interaction energies (Figure S1, Supporting Information File 1) measured with the N_2_O tip (outer C–C bond, 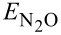 ≈ −156 meV, and hollow site, 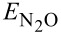 ≈ −167 meV) are substantially greater in comparison to the values measured by the CO tip (outer C–C bond, *E*_CO_ ≈ −43 meV, and hollow site, *E*_CO_ ≈ −75 meV). This difference can be understood as a result of stronger electrostatic interaction of the molecule with the N_2_O tip, which is consistent with the DFT calculations of the two different tip terminations.

## Conclusion

We have investigated the behavior of N_2_O molecules on the surface of Au(111) and determined that they adsorb parallel to the surface, forming typical triangular clusters. We were able to readily functionalize a metallic tip with a single N_2_O molecule by picking it up from the Au(111) substrate and demonstrated that the functionalization of the tip can be achieved even when N_2_O is co-adsorbed on the surface with other species, in this case FePc molecules. We evaluated the performance of the N_2_O tips in submolecular imaging of FePc and site-specific Δ*f*(*z*) spectroscopies. We reproducibly achieved a resolution qualitatively equivalent to the resolution otherwise routinely observed with CO tips, distinguishable by a noticeable asymmetry and higher interaction energies, indicative of a bent adsorption geometry of the N_2_O on the tip and more electrostatic charge relative to CO. These observations were corroborated by DFT calculations.

## Methods

### Experimental

Experiments were carried out in an ultra-high vacuum STM/AFM system (Createc) operated at 5 K. The Au(111) sample (Mateck) was cleaned by repeated cycles of sputtering (1 keV) and subsequent annealing to 600 °C. FePc molecules (Sigma Aldrich, evaporation temperature ca. 250 °C) were directly evaporated onto a clean Au(111) surface at room temperature. N_2_O was adsorbed onto the Au(111) surface at temperatures below 12 K with exposures of 0.5–1.7 L. AFM measurements were performed with a qPlus sensor (resonance frequency ca. 30 kHz; *k* ≈ 1800 N/m), using an oscillation amplitude of 50 pm. Prior to functionalization, the Pt tip was repeatedly indented into the Au(111) substrate several nanometers deep for sharpening and coating with Au. Experimental data were analyzed using WSxM software [40]; all models were visualized using Vesta software [41].

### DFT calculations

We performed density functional theory calculations using the FHI-AIMS code [42] to study the interaction of N_2_O with the Au(111) surface. We have used a 6 × 6 supercell, composed of three Au layers to represent the Au(111) surface. Both a single molecule and trimer clusters were initially placed on the surface according to experimental findings. The structural optimization of the slab was carried out, except for the two bottom Au layers, until the remaining atomic forces and the total energy were found to be below 10^−2^ eV/Å and 10^−5^ eV, respectively. A Monkhorst–Pack grid of 3 × 3 × 1 was used for integration in the Brillouin zone.

DFT calculations were performed at the GGA-PBE level including the Tkatchenko–Scheffler treatment of the van der Waals interactions [43]. The scaled zeroth-order regular approximation [44] was applied to take into account the relativistic effects. The total density and the Hartree potential were calculated to determine the electronic interactions between the surface and the molecules.

AFM images were simulated based on the probe-particle model [32,45], which takes into account van der Waals (vdW) and electrostatic interactions between the tip and the sample. The calculations were performed varying the effective charge of the probe particle in order to obtain the best possible agreement between the experimental findings and the simulated AFM images. The lateral stiffness was set to *k* = 0.25 N/m. The correlation of the experimental evidence and theory permit us to understand the nature and origin of the chemical contrast.

## Supporting Information

File 1 Additional computational data.
