# Supplementary material for: Nitrous oxide as an effective AFM tip functionalization: a comparative study
Source: Beilstein J Nanotechnol. 2019 Jan 30;10:315–21. doi: 10.3762/bjnano.10.30 (PMC6369984; doi:10.3762/bjnano.10.30)
Supplement: File 1 — Additional computational data. [file Beilstein_J_Nanotechnol-10-315-s001.pdf]

BEILSTEIN JOURNAL OF NANOTECHNOLOGY

Supporting Information

for

Nitrous oxide as an effective AFM tip functionalization: a comparative study

Taras Chutora, Bruno de la Torre, Pingo Mutombo, Jack Hellerstedt, Jaromír Kopeček, Pavel Jelínek and Martin Švec

Beilstein J. Nanotechnol. **2019,** *10,* 315–321. doi:10.3762/bjnano.10.30

Additional computational data

License and Terms: This is a supporting information file under the terms of the Creative Commons Attribution License (http://creativecommons.org/licenses/by/4.0). Please note that the reuse, redistribution and reproduction in particular requires that the authors and source are credited.
The license is subject to the *Beilstein Journal of Nanotechnology* terms and conditions: (https://www.beilstein-journals.org/bjnano)

Calculated force and energy

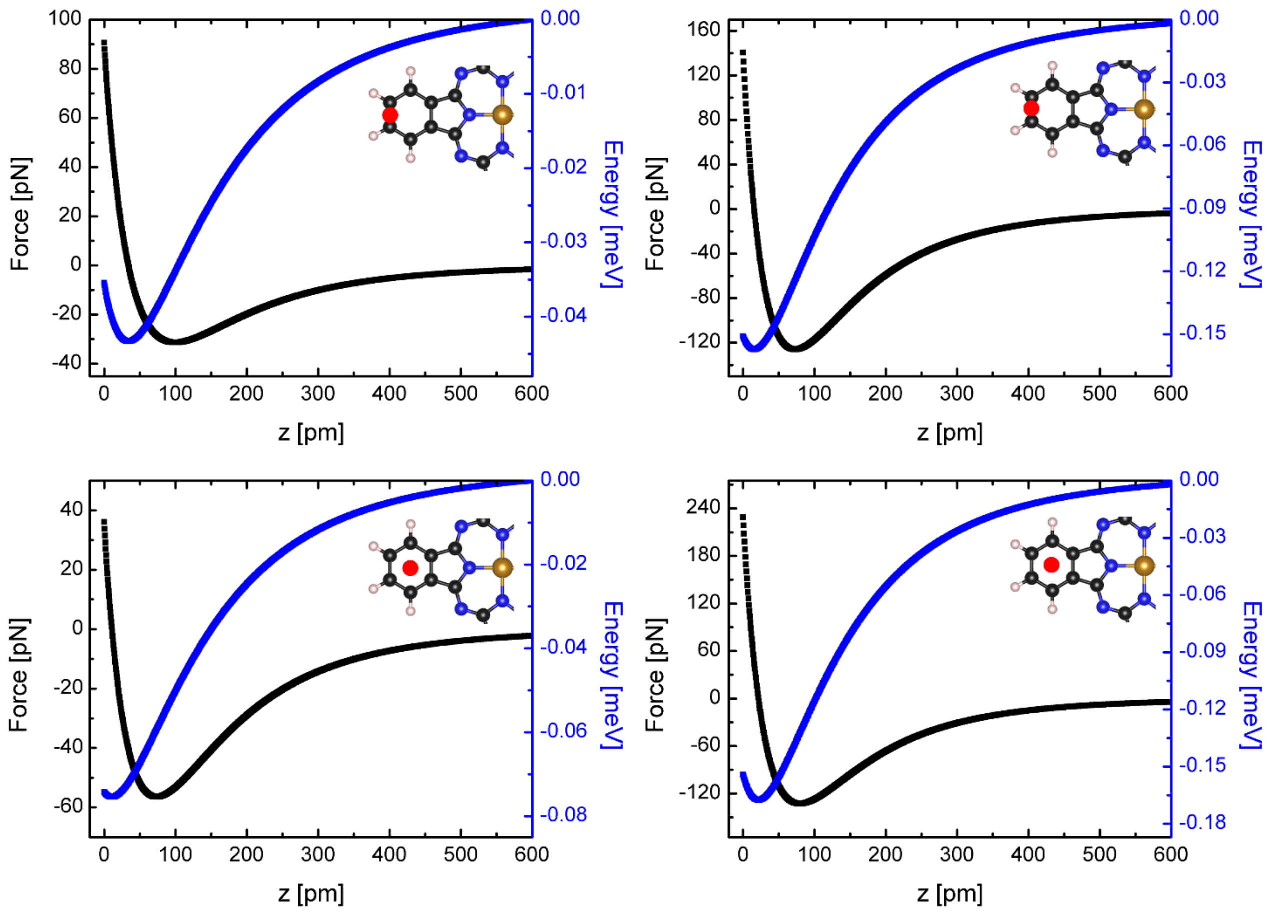

Figure S1: Deconvoluted force and energy from the experimentally obtained frequency shift as a function of the tip–sample distance for CO (left side) and N_2O tips (right side). The long-range component measured on bare Au(111) was subtracted prior to calculation from the total interaction curve. The measurements were acquired above an outer C–C bond and above a hollow site of a peripheral benzene of the FePc molecule (position indicated with a red dot in the inset of each plot). The force and energy were calculated using the Giessibl method [1].

References

[1] Giessibl F. J. *Appl. Phys. Lett*. **2001,** *78,* 123–125. doi:10.1063/1.1335546
